# The greatest Dengue epidemic in Brazil: Surveillance, Prevention, and Control

**DOI:** 10.1590/0037-8682-0113-2024

**Published:** 2024-09-20

**Authors:** Rodrigo Gurgel-Gonçalves, Wanderson Kleber de Oliveira, Julio Croda

**Affiliations:** 1 Universidade de Brasília, Faculdade de Medicina, Núcleo de Medicina Tropical, Laboratório de Parasitologia Médica e Biologia Vetores/Programa de Pós-Graduação em Medicina Tropical, Brasília, DF, Brasil.; 2 Centro Universitário do Planalto Central Apparecido dos Santos, Faculdade de Medicina, Brasília, DF, Brasil.; 3 Direção Técnica de Ensino e Pesquisa, Hospital das Forças Armadas, Brasília, DF, Brasil.; 4 Universidade Federal de Mato Grosso do Sul, Faculdade de Medicina, Campo Grande, MS, Brasil.; 5 Yale School of Public Health, Department of Epidemiology of Microbial Diseases, New Haven, CT, USA.; 6 Fundação Oswaldo Cruz, Campo Grande, MS, Brasil.

**Keywords:** Dengue, Epidemiology, Mosquito control, Prevention, Brazil

## Abstract

In this review, we discuss dengue surveillance, prevention, and control measures in Brazil. Data on dengue epidemics between 2000 and 2024 indicates an increase in the number of dengue cases and deaths. Global climate change is a key driver of this growth. Over the past 25 years, nearly 18 million Brazilians have been infected with the dengue virus, and the highest number of dengue cases in Brazil's history is projected to reach 2024. Dengue mortality in Brazil increased geographically over time. As of June, there were approximately 6 million probable cases and 4,000 confirmed deaths in Brazil, which represents the greatest dengue epidemic to date. Several technologies have been developed to control *Aedes aegypti*, including the deployment of *Wolbachia*-infected mosquitoes, indoor residual spraying, sterile insect techniques, and mosquito-disseminated insecticides. The Ministry of Health recommends integrating these technologies into health services. Brazil is the first country to incorporate the Takeda vaccine into its public health system, and the Butantan vaccine is currently undergoing Phase 3 clinical trials. Increasing the vaccination coverage and implementing novel *Ae. aegypti* control technologies could reduce the number of dengue cases in Brazil in the coming years. Community activities such as home cleaning and elimination of potential mosquito breeding sites, facilitated by social media and health education initiatives, must continue to achieve this reduction. Ultimately, a multisectoral approach encompassing sanitary improvements, mosquito control, vaccination, and community mobilization is crucial in the fight against dengue epidemics.

## INTRODUCTION

Dengue is an infectious disease caused by four related viruses that are transmitted by *Aedes* mosquitoes. Approximately half of the global population is at risk of dengue infection, with 100-400 million people developing deadly fever and 40,000 deaths from dengue each year^1^. *Aedes aegypti* is increasingly present in urban areas^2^ ^-^ ^4^ and the number of dengue cases in the Americas has increased from 1.5 million in the 1980s to 16 million in the decade 2010-2019^5^. In Brazil, the first epidemic began in 1986^6^ and a rapid expansion of dengue has been observed recently^7^. The four serotypes of the virus circulate simultaneously, and mosquitoes are present in every region of the country^8^. Between 2008-2019, approximately 6,429 Brazilians died of dengue^9^. Based on epidemiological data from June 2024, there were 10 million probable cases of dengue and approximately 5,000 confirmed deaths reported in the Americas^5^ and Brazil is currently experiencing its greatest dengue epidemic to date. As of June 15th, there were approximately 6 million probable cases and 4,000 confirmed deaths^10^.

Dengue epidemics have multiple causes, including rising temperatures and rainfall due to climatic changes, inadequate sanitation, insufficient numbers of health workers, poor efficacy of government interventions to control *Ae. aegypti*, discontinuity of activities throughout the year, population difficulty in eliminating domestic breeding sites, the resistance of mosquitoes to insecticides, and the presence of four serotypes circulating simultaneously in endemic countries that favor reinfection^7^. Furthermore, *Ae. aegypti* females can lay eggs in many different water-holding container habitats with different degrees of cleanliness, resist drought, adapt to warming climates and increasing altitudes^11^. However, new technologies have been developed for surveillance, control, and prevention of dengue^12^ ^-^ ^19^. Integrated vector control methods, including mosquito control technologies and vaccines, offer a critical boost to combating dengue epidemics. However, active public and government engagement must complement these efforts. This requires educating and mobilizing the population to maintain cleanliness in their homes and eliminate potential breeding sites. Continuous outreach through social media, community-based initiatives like "D-Day against Dengue,” and creative public awareness campaigns are essential to this multisectoral approach^16^ ^,^ ^17^.

We reviewed documents published on dengue in Brazil over the last 25 years, updated the epidemiological scenario, and provide a critical analysis of strategies for the surveillance, prevention, and control of the disease in Brazil. Our narrative review^20^ provides a critical overview of these subjects and their perspectives regarding Brazil. We included epidemiological data from the Notifiable Diseases Information System (SINAN, accessible at: ftp://ftp.datasus.gov.br/dissemin/publicos/SINAN/), Ministry of Health of Brazil. The epidemiological scenario was based on data from the 21st century. We excluded data prior to 2000 because they were not available in the public database. Our search included the terms “dengue” and “Brazil” and all types of references related to the topics (original articles, reviews, commentaries or opinion pieces in PubMed, gray literature, reports, or digital media in English, Portuguese, and Spanish) were considered. This review also incorporated additional references identified through manual search. This review describes the increase in dengue cases and deaths in Brazil and the geographical expansion of dengue cases and deaths by age group. Considering that the classification of Dengue cases has changed over the years in Brazil, we used the following categorization: dengue A which includes the old definition of classic dengue and the current definition of dengue; Dengue B, which includes dengue with complications, dengue with alarm signs, and dengue hemorrhagic fever types I and II and Dengue C, which includes severe dengue and dengue shock syndrome, which are dengue hemorrhagic fever types III and IV. These definitions are available in the epidemiological and health surveillance guides as well as in the notification forms and data dictionary of the SINAN. Global climate change has been highlighted as one of the main causes of this growth, and new technologies for controlling *Ae. aegypti* are described. In addition, advances in dengue vaccines and prospects for surveillance, control, and prevention are outlined. 

## SURVEILLANCE

**FIGURE 1:**
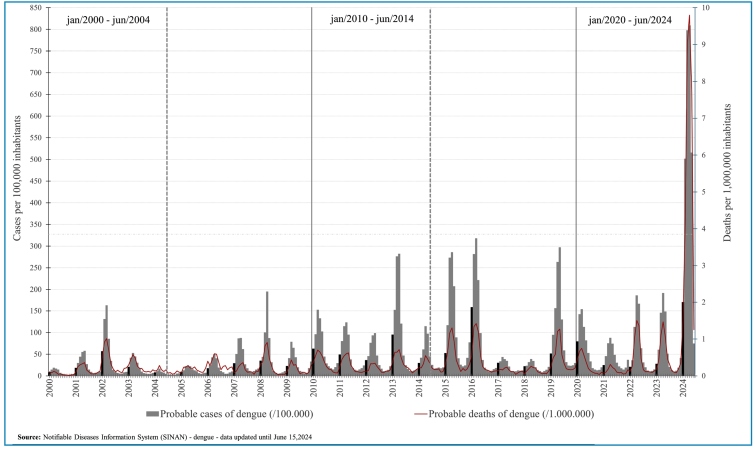
Dengue epidemics in Brazil between 2000 and 2024. The historical series shows probable cases of dengue/100,000 (gray) and probable deaths of dengue/1000,000 inhabitants (red) recorded by month in each year. The small black bars indicate the probable cases of dengue/100,000 in January for each year. The longer black bar indicates the decades. The vertical dotted lines mark the first 4.5 years of each decade.

Dengue has been a notifiable disease in Brazil since 1961 (Decree nº 49.974-A, January 1961). However, electronic registration through the Notifiable Diseases Information System was only implemented in 1993. Currently, the system consolidates records across the country into a centralized database at the federal level. Dengue cases without complications are transferred weekly, whereas severe cases and deaths must be transferred within 24 h of detection by the health service. Brazil is currently experiencing an unprecedented dengue epidemic in 2024. The historical series of dengue epidemics in Brazil between January 2000 and June 2024 (Figure 1) showed that until 2012, the number of cases was close to or below 200,000. An epidemic with more than 400 cases/100,000 only occurred in 2024. The highest number of dengue deaths/1000,000 inhabitants in the history of the disease in Brazil is projected to reach 2024. We analyzed epidemiological data from the first 4.5 years of each decade to establish equivalence with the 2020-2024 period. Between January 2000 and June 2004, 2,073,194 probable dengue cases were reported in Brazil. In the same period of the following decade, between January 2010 and June 2014, there were 6,260,684 probable cases, representing a percentage increase of approximately 202% compared to the previous period. In the most recent decade, between January 2020 and June 2024, 11,236,426 probable cases of dengue were recorded, representing a percentage increase of about 442% compared to the period 2000 to 2004 and an increase of about 80% compared to the period 2010 to 2014. These data highlighted the progression of dengue during these periods (Figure 1, Supplementary file 1).

**FIGURE 2:**
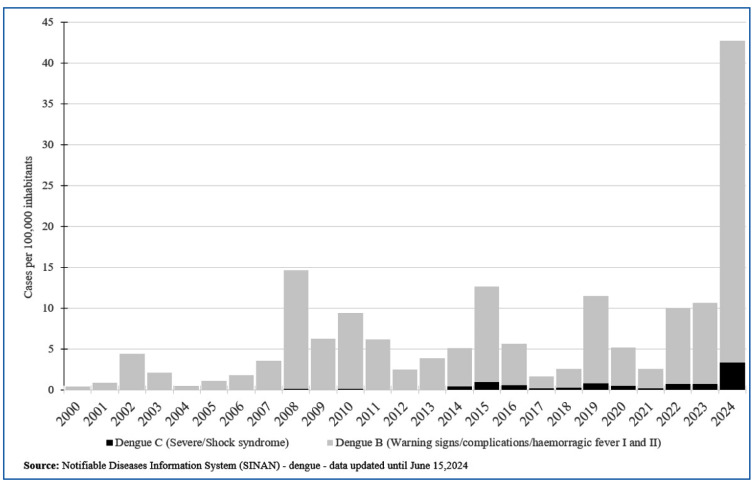
Number of severe/shock syndrome (black bars) and dengue with warning signs and other complications (gray bars) reported in Brazil between 2000 and 2024.

**FIGURE 3:**
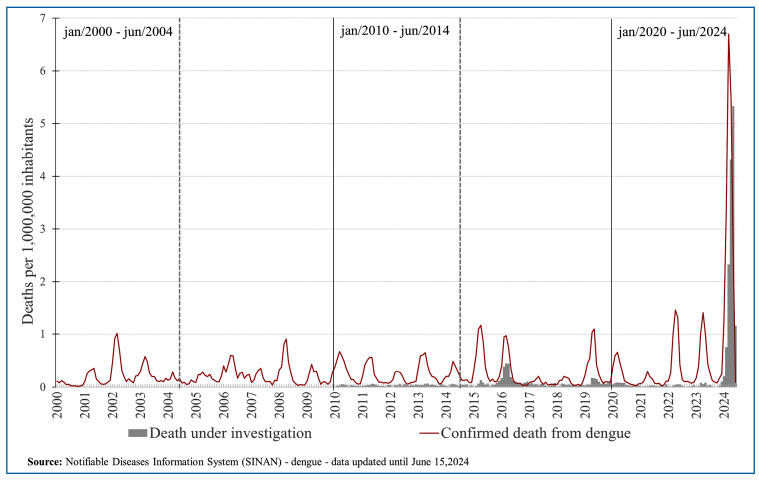
Number of dengue deaths under investigation (gray) and confirmed death from dengue (red)/1,000,000 inhabitants in Brazil between 2000 and 2024. The longer black bar indicates the decades. The vertical dotted lines mark the first 4.5 years of each decade.

There is a marked peak in dengue cases with warning signs by 2024 (Figure 2). The number of dengue deaths under investigation and confirmed deaths from dengue per 1,000,000 inhabitants in Brazil will increase significantly by 2024 (Figure 3).

**FIGURE 4:**
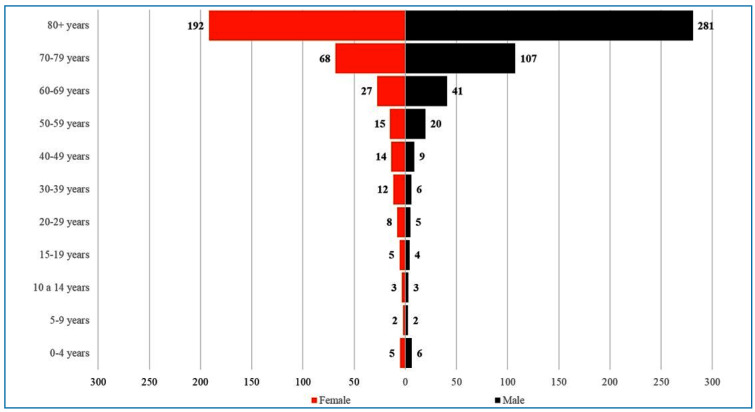
Dengue incidence rate reported in Brazil in 2024, until June. The bars represent the number of deaths by age/1,000,000 inhabitants and the color represents the sex (male: black, female: red). Source: Notifiable Diseases Information System (SINAN).

Preliminary data for 2024 up to June 15th shows ~ 6 million probable cases of dengue, five times the number in the same period in 2023. The average incidence of dengue in the first three months of 2023 and 2024 is 69 and 187 cases per 100,000 inhabitants, respectively. Additionally, in the first six months of 2024, there were approximately 80,000 cases with warning signs and other complications and 6,791 severe cases of dengue (Supplementary file 1), which is 5-6 times higher than the numbers observed in the same period in 2023. Additionally, the death rate was seven times higher in June 2024 than that in the same period in 2023. These data indicate a greater impact of the 2024 dengue epidemic. The historical record of dengue cases with warning signs and other complications, dengue deaths, or deaths under investigation shows that 2024 has reached much higher numbers than all other dengue epidemics in Brazil (Figures 1-3). We evaluated deaths under investigation from previous years to show that not all cases were closed because some municipalities do not have a committee to investigate deaths. Approximately 90% of the deaths under investigation are already dengue deaths and have not been reclassified, owing to operational limitations at the municipal level. During the latest epidemic (until June 2024), the number of dengue deaths was higher among the elderly (Figure 4). This differs from the pattern observed in other epidemics, where severe dengue cases were higher among children aged 6-10 years^21^. These data suggest that targeting both the elderly and young age groups could help reduce severe disease outcomes.

Dengue serotypes have spread throughout Brazil^7^, and the predominance of DENV-2 is possibly responsible for increased dengue mortality^22^ ^,^ ^23^. DENV-2 has emerged and caused epidemics in severe cases and hospitalizations^24^ ^,^ ^25^. Severe dengue includes multiple organ failure and renal involvement, and may be associated with increased mortality^25^. Experimental data show an increase in kidney weight in mice infected with DENV-2^26^, and epidemiological data show that severe dengue is associated with DENV-2^27^. Dengue epidemics occur in different regions of the country, and cases of DENV-1, DENV-2, and DENV-3 have been reported in all states. DENV-4 cases were identified in the northern region and Rio de Janeiro^6^. 

**FIGURE 5:**
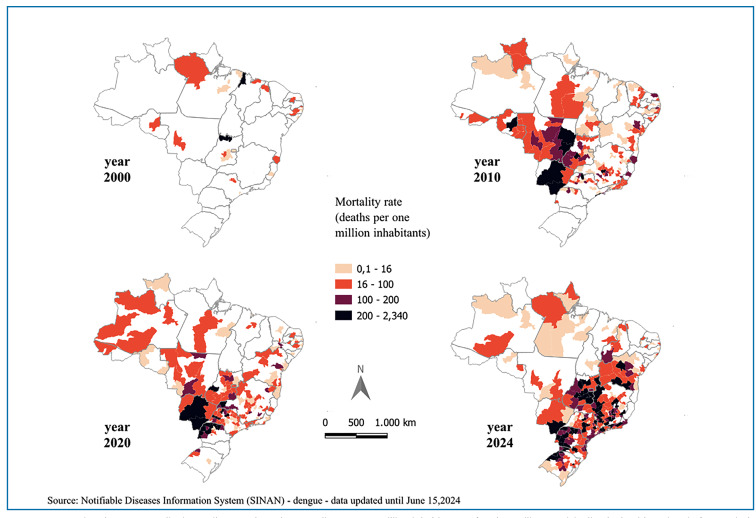
Mapping dengue mortality in Brazil. Maps show the mortality rates (/1 million inhabitants) of each Brazilian municipality (by health region) in four periods (2000, 2010, 2020 and 2024). The color gradient represents the variation in the mortality rate. These maps were created using QGIS (version 3.36.1) and SINAN mortality data until June 2024.

By 2024, emergency decrees were issued across 11 federal units, affecting 602 municipalities. Figure 5 displays the mortality rates (per 1 million inhabitants) of each Brazilian municipality organized by health region for four years (2000, 2010, 2020, and 2024). The data reveal an increase in dengue mortality in Brazil over time. In 2000, the mortality rates were higher in more isolated healthy regions, excluding the south. From 2010 onwards, there was an expansion to the east and west of the country. Finally, in 2024, expansion to the south will become evident, as has already been observed^7^. Additionally, a higher mortality rate was observed in states such as Goiás, the Federal District, and Minas Gerais. 

Mapping the dengue incidence and mortality rates in the country is crucial for targeting clinical management policies and reducing dengue hospitalizations and deaths in the future^6^ ^,^ ^28^ ^-^ ^30^. Brazil is a large country, and dengue occurs differently in various regions, states, and municipalities^31^ ^,^ ^32^. Recurrent annual dengue epidemics are currently common in less populated areas of Brazil that are free of dengue transmission^6^. There has been an increase in dengue deaths in Brazil since 1986^7^ and our study shows that this trend has continued in recent years, with the worst year being 2024. In recent years, the central-west region has exhibited the highest dengue incidence and mortality rates. Epidemiological data indicated that municipalities that experienced an outbreak in the past were twice as likely to experience subsequent outbreaks^33^. Historically, the western Amazon, southern region, and northern coast of Brazil have been considered geographical barriers to dengue transmission. However, the historical series show that practically no area is protected against dengue. Climate change, mosquito adaptation, and growing urbanization with precarious housing and sanitation conditions in cities may also explain the higher occurrence of *Aedes* and incidence of dengue over the years^11^ ^,^ ^33^ ^-^ ^37^. The data revealed the spread of dengue to smaller municipalities in central-south Brazil, far from more urban centers, while larger cities were classified as having sustained transmission, which varies across Brazilian states, suggesting that other factors contribute to the rising cases in smaller towns^38^. 

Dengue fever has historically brought about substantial costs and societal impacts in Brazil^39^. Another critical aspect of the current public health challenge is the concurrent CHIKV outbreak, which has complicated the epidemiological landscape. This misclassification underscores the complexity of managing arbovirus outbreaks and the importance of accurate diagnostic capabilities to distinguish similarly presenting diseases. Moreover, the Brazilian Ministry of Health should improve the protocol for death confirmation and timely surveillance to reduce delays in death investigations. This can be achieved by strengthening the surveillance team to complete the investigation in a timely manner. Such intricacies highlight the need for a more nuanced understanding of the severity of an outbreak and necessitate adaptive strategies for disease management and response. 

The impact of climate change on the global spread of dengue fever, particularly in Brazil, is a critical issue requiring comprehensive understanding and action^40^. Climate change and its associated phenomena, such as rising temperatures, altered precipitation patterns, and extreme weather events, have profound implications for the life cycle and distribution of *Aedes* mosquitoes, the primary vectors of the dengue virus^41^. Warmer temperatures can accelerate mosquito breeding, increase biting rates, and shorten the viral incubation period within mosquitoes, potentially leading to higher transmission rates of dengue fever^42^. For instance, climate and weather alterations, including temperature, rainfall, humidity, and El Niño, have been identified as critical factors affecting the reproduction, survival, and geographic distribution of mosquitoes, ultimately influencing their capacity to transmit pathogens^33^ ^,^ ^43^. Dengue surveillance has been supported by nowcasting and forecasting data^2^ ^-^ ^4^ ^,^ ^16^ based on climate data, and mosquito and dengue distribution forecasts.

In Brazil, studies have utilized data mining techniques to investigate the recent expansion and exacerbation of the dengue incidence, revealing that prolonged temperature anomalies, urbanization, and previous circulation of the virus were significant contributors to the increased incidence in the central region of the country. The occurrence of dengue outbreaks was positively associated with the number of months per year with favorable temperature conditions for *Aedes* mosquitoes^33^. Areas at higher altitudes, once natural barriers to dengue transmission, have now become hotspots for the disease, demonstrating the changing dynamics of disease distribution in response to climatic shifts^40^. This expansion towards new areas, including higher altitudes and latitudes, underscores the influence of climate change on altering the landscapes of vector-borne disease risks, necessitating adaptive strategies for public health planning and vector control efforts^33^. 

## VECTOR CONTROL

The World Health Organization (WHO) is urging the integration of new technologies into health services, including the stratification method, *Wolbachia*-infected mosquito deployment method^44^, mosquito-disseminated insecticide strategy^45^, intradomiciliary residual spraying^46^, and sterile insect techniques, which are recommended by scientific evidence^12^ and the Brazilian Ministry of Health^47^. 

Stratifying areas based on epidemiological and environmental data is the key to organizing dengue surveillance and control efforts. This approach considers the seasonal patterns of the disease and helps to optimize the use of local resources. Research has highlighted the importance of continuous disease monitoring, timely data analysis, and prompt action as essential components to effectively combat dengue in Brazil. ArboAlvo^48^ is an example of a stratification analysis based on socioenvironmental indicators recommended by the Brazilian Ministry of Health. 

Releasing *Ae. aegypti* infected with *Wolbachia* is a promising strategy. Over time, the proportion of mosquitoes carrying *Wolbachia* tends to increase until the entire mosquito population was infected. *Wolbachia* reduces the lifespan of mosquitoes by 50% and inhibits the development of dengue virus within them^49^. This technology has been successful in reducing dengue incidence in Australia^50^ and Indonesia^44^. Few randomized trials have evaluated the control methods for *Ae. aegypti*, without the use of the gold standard endpoint for virologically confirmed dengue. The results of a single trial conducted in Yogyakarta, Indonesia demonstrated the efficacy of *Wolbachia*-infected *Ae. aegypti* mosquitoes to control dengue transmission. The study demonstrated the significant potential of the *Wolbachia* method in public health, with a 77% reduction in virologically confirmed dengue cases and an 86% reduction in hospitalizations for dengue^36^. This intervention was equally effective against all four dengue serotypes, indicating its robustness and protective efficacy. In Rio de Janeiro, Brazil, the release of *Wolbachia*-infected *Ae. aegypti* resulted in 38%^51^ and 69%^52^ reduction in the incidence of dengue, respectively. Establishing stable *Wolbachia* strains in geographically diverse urban settings, such as Rio de Janeiro, appears to be more challenging than in other locations^51^. The release program requires specialized infrastructure in the municipalities, but it is likely to be a cost-effective strategy in the Brazilian context, considering that alternative scenarios have shown a favorable return on investment with a positive benefit-cost ratio^53^. 

The mosquito-disseminated insecticide strategy is a low-cost technology for controlling *Ae. aegypti* breeding sites. The technique is based on the deployment of dark plastic pots filled with water (dissemination station, DS hereafter), in which a larvicide (an insect juvenile hormone analog, such as pyriproxyfen) is impregnated in a cloth that covers the pot internally. When mosquitoes land on DS, the larvicidal particles stick to their bodies and are transferred by the mosquitoes themselves to other larval habitats^54^ ^,^ ^55^. DS has yielded promising results in trials at the scales of neighborhoods^45^ and towns^56^. Garcia et al.^57^ using a cluster-randomized controlled trial with 16 months of field data and a rigorous statistical modeling strategy and showed that DS can significantly reduce adult mosquito densities by 66%. Moreover, DS can block the transmission of mosquito-borne viruses^56^. Because of the low cost and elimination of hidden, difficult-to-access breeding sites, DS should be adopted by control services, as recommended by the Ministry of Health, as it is an easy method to be executed by health agents who deal with vector control. This method should be used along with other vector control initiatives, and future studies should evaluate the effectiveness of DS on the incidence of dengue^47^.

Intradomiciliary residual spraying involves the application of residual insecticides to the interior walls of buildings and is commonly used to control vectors of malaria, Chagas disease, and leishmaniasis. When mosquitoes land on walls, they are exposed to insecticides and die. Previous studies demonstrated that this method is effective against *Ae. aegypti* despite difficulties in training, equipment calibration, insecticide costs, and mosquito resistance to insecticides^46^ ^,^ ^58^ ^,^ ^59^. The Brazilian Ministry of Health recommends its use before the start of the epidemic period, especially in buildings with many people (schools, health units, and community centers)^47^. Currently, the insecticide Fludora® Fusion is recommended for indoor residual spraying against the *Ae. aegypti* mosquito. According to a technical report, the Ministry advises that this residual application should be conducted every two months, and periodic evaluation and monitoring efforts should be implemented to assess the efficacy of this control activity^47^.

The use of sterile insects is another strategy recommended by the Ministry of Health. This technique is based on the release of *Ae. aegypti* sterile males in an area, with the objective of promoting the copulation of these males with females in the area and making the offspring unviable. Despite advances in the development of this technique and examples of successful application^60^, its expansion to larger areas depends on optimizing protocols for handling, transporting, and releasing male mosquitoes^61^. This tool is indicated in areas where mosquitoes are highly resistant to insecticides and periodic releases of males are available^47^.

Finally, control of *Ae. aegypti* must be planned according to the local health structure and based on surveillance data that can indicate the best control strategy, or even the use of combined strategies, including the eco-social context. There were no silver bullets to control *Ae. aegypti*. Brazil has 5,700 municipalities with different socioeconomic, geographic, and climatic characteristics; even within a single municipality, there are variations. In addition to inter- and intra-urban heterogeneity, transmission dynamics are influenced by the patterns of population mobility and the large number of asymptomatic infected individuals circulating, which enhance mosquito infection and dengue transmission. Therefore, analysis of entomological and epidemiological data over a short period is crucial. Moreover, stratification analysis is important for selecting the appropriate strategy or best combination of strategies for each municipality^62^. In this integrated strategy, the Ministry of Health is the key to fostering the intersectoral actions needed to plan, finance, and implement priority activities outlined in municipal control, as proposed in the multisectoral approach to the prevention and control of infectious and vector-borne diseases^63^ ^,^ ^64^. 

## PREVENTION

Development of a dengue vaccine has been a noteworthy endeavor in the field of infectious diseases. Sanofi's Dengvaxia was the first study to make significant progress in providing partial protection against four dengue virus serotypes^65^. Its development represented a significant milestone in dengue prevention. However, the effectiveness of the vaccine varies by serotype, and was later found to be associated with severe dengue in seronegative individuals, thereby limiting its widespread use^13^. The potential consequences of a fully effective vaccine for all four serotypes are substantial and promising for reducing the global burden of dengue, provided that it can overcome safety challenges and has broad serotype efficacy.

The Takeda vaccine candidate, TAK-003, demonstrated its potential through its tetravalent formulation. Early trial results were favorable, suggesting protection against multiple serotypes and a satisfactory safety profile^14^. The vaccine has received approval from Indonesian, European, and Brazilian regulatory agencies, and the World Health Organization (WHO) Strategic Advisory Group of Experts on Immunization (SAGE) group has recommended its use in areas with high disease prevalence in children aged 9-16 years, with vaccination initiated 1-2 years prior to peak incidence in this age group. In December 2023, Brazil became the first country to approve the incorporation of the vaccine into its public health system, with 8·5 million doses expected to be available by 2024. TAK-003 shows high efficacy against symptomatic cases and hospitalizations, particularly against dengue serotypes 1 and 2, although additional data on serotypes 3 and 4 in seronegative individuals are still pending^14^. A study was conducted in Dourados, Brazil, evaluating the influence of a planned mass vaccination program against dengue, aiming to immunize 100,000 individuals aged 4-60 years from January to August 2024. This study provides information on the impact of the mass vaccination campaign in a city with a population of 243,368. Furthermore, this study aimed to determine the effectiveness of the vaccine against dengue. If there are sufficient cases of autochthonous transmission of dengue serotypes 3 and 4 in Dourados over the next 5 years, the researchers plan to assess the vaccine's effectiveness against specific serotypes (JC, personal communication).

The Butantan Institute and National Institutes of Health (NIH) have collaborated to develop a live-attenuated vaccine candidate that could elicit a robust immune response against all four dengue serotypes. The findings of a phase 3 trial of the Butantan-Dengue Vaccine (Butantan-DV), a single-dose vaccine, have recently been published. The trial was conducted across various locations in Brazil and aimed to assess the vaccine's efficacy and safety in a diverse demographic spanning children to adults aged 2-59 years. The trial participants were stratified by age and randomized to receive either Butantan-DV or a placebo, with the primary objective of evaluating the vaccine's efficacy against symptomatic, virologically confirmed dengue of any serotype after 28 days of vaccination and to monitor safety up to 21 days post-vaccination. Over a 2-year follow-up period, Butantan-DV demonstrated a significant efficacy rate of 79.6%, with notable efficacy across different age groups and dengue serostatuses at baseline. The vaccine's efficacy was particularly strong against DENV-1 and DENV-2 serotypes, with rates of 89.5% and 69.6%, respectively^15^. Additionally, the vaccine was well tolerated, with adverse events more commonly reported in the vaccine group than in the placebo group within the first 21 days post-vaccination, but without serious safety concerns. These findings underscore the potential of Butantan-DV as a single-dose vaccine for significantly reducing the burden of dengue across a broad age range, marking a significant advancement in the global fight against dengue.

Although the development of a pan-serotype dengue vaccine has made significant progress, significant obstacles remain. Two of the most promising dengue vaccine candidates have limited efficacy and safety data for serotypes 3 and 4. Additionally, the risk of antibody-dependent enhancement (ADE), observed in Sanofi's Dengvaxia poses a challenge for the deployment of new vaccine candidates. It is crucial to recognize that only real-world data on the effectiveness and safety of both vaccines will fill this gap. Ensuring the absence of ADE in new vaccines is essential for the global acceptance of new dengue vaccines. 

## CONCLUSIONS AND OUTLOOK

Since 1986, Brazil has experienced frequent dengue epidemics, resulting in significant social and economic impact. A historical series of dengue epidemics between 2000 and 2024 indicated an increase in the number of dengue cases and deaths. Over the past 25 years, nearly 18 million Brazilians have been infected with the dengue virus, and the highest number of dengue cases in Brazil's history is projected to reach 2024. Data show that dengue mortality in Brazil has expanded geographically over time. Approximately 17,000 Brazilians have died of dengue in the last 25 years. As of June 2024, there have been approximately 6 million probable cases and 4,000 confirmed deaths in Brazil, representing the greatest dengue epidemic to date, with the co-circulation of different dengue serotypes. Global climate change is one of the main factors contributing to this growth. Viruses and mosquitoes have expanded their distribution throughout the country, causing epidemics in new areas where health systems are unprepared to handle a high number of cases. The Ministry of Health has promoted policies to manage dengue epidemics, including the introduction of the NS1 rapid test, establishment of a Center of Emergency Operations (CEO), financial transfers to support states and municipalities in arbovirus surveillance and prevention activities, and investments in innovation for dengue control^66^ ^-^ ^68^. The CEO was established by the Ministry of Health in February 2024 to improve planning and coordinate responses against arboviruses in an integrated and articulated manner with the states and municipalities throughout the country^68^. However, the CEO faces challenges in implementation in the municipalities due to the constant change of professionals and insufficient training of health teams to implement clinical management guidelines. Brazil needs to move towards more anticipated surveillance actions by applying nowcasting and forecasting models, considering that dengue is a seasonal disease, and healthcare should be prepared before the epidemic begins. An additional limitation regarding the impact of dengue fever in Brazil is the quality of diagnostic procedures, which encompasses inaccuracies in the identification of arboviral infections, as well as the recognition of the clinical signs and symptoms^8^ ^,^ ^67^. During significant dengue outbreaks, such as the 2024 epidemic, doctors may face substantial pressure to diagnose dengue cases. This could have led to an inflated number of reported cases, particularly in smaller municipalities, where many healthcare providers lack the necessary experience to accurately diagnose the disease. Consequently, training programs are recommended to improve the diagnosis and clinical management of dengue in the healthcare systems of the affected municipalities.

Our appraisal provides an updated synthesis of new technologies for the control *Ae. aegypti* in Brazil. Effective control is limited by the difficulty in identifying and controlling mosquito breeding sites, which is worsened by the resistance of mosquitoes to most insecticides. Most dengue mosquito breeding sites are situated within household premises, underscoring the need to collaborate with residents for effective control measures. Additionally, surveillance with active engagement of health workers throughout the year is critical for successful dengue management. These surveillance efforts should be supported by public policies aimed at enhancing professional development, communication, mobilization, and education of the population. Recently, new technologies have been developed to control *Ae. aegypti* and are now recommended by the Brazilian Ministry of Health. Integrating these technological solutions into the healthcare systems of Brazilian municipalities, tailored to their specific circumstances, represents the next critical challenge. While these technical interventions may assist in controlling future dengue outbreaks, their effectiveness depends on various external factors, including climate change, inadequate sanitation infrastructure, the introduction of new dengue virus serotypes in different regions, and the implementation of appropriate public health policies at the national, state, and municipal levels. Given the varying environmental, sociodemographic, and healthcare scenarios across municipalities in Brazil^38^ ^,^ ^69^, it is crucial for managers to determine, with support from state and federal levels, which strategies to implement for dengue control (e.g., *Wolbachia* method, mosquito-disseminated insecticide strategy, and others recommended by the Ministry of Health). Otherwise, dengue control will continue to be based on traditional measures that have not been able to reduce the growth of mosquito populations and, consequently, dengue transmission. It is important to emphasize that the monitoring and control methods described here depend on the services provided by a group of health agents that must be expanded and valued. We believe that continuous education is needed to raise awareness of the importance of keeping residential environments free of *Ae. aegypti* breeding sites. It is important to note that most breeding sites were located in homes. To intensify the mobilization of the population, social media and collective actions, such as D-Day against Dengue, 10 minutes against dengue, and creative advertising campaigns, can be employed. Moreover, real-time mapping, social media platforms, such as DengueChat, and alerts to health professionals in at-risk areas are promising strategies for dengue surveillance and control^70^ ^,^ ^71^.

Dengue vaccine development has made notable strides and has led to significant advancements in public health efforts to mitigate the global burden of dengue fever. This journey began with Sanofi's Dengvaxia, the first vaccine to offer partial protection against the four dengue virus serotypes. Despite its groundbreaking achievements, the varied efficacy of Dengvaxia among serotypes and its association with severe dengue in seronegative individuals limits its universal application. Takeda's TAK-003 emerged as a promising candidate, showcasing protection across multiple serotypes and a favorable safety profile, receiving endorsements from international and national regulatory bodies. The incorporation of TAK-003 into Brazil's public health system and planned mass vaccination programs represents a pivotal moment in dengue prevention efforts, especially against serotypes 1 and 2, with ongoing investigations of its efficacy against serotypes 3 and 4. Moreover, collaboration between the Butantan Institute and NIH has led to the development of the Butantan-Dengue Vaccine (Butantan-DV), a single-dose, live-attenuated vaccine candidate. Early phase 3 trial results in Brazil demonstrated its significant efficacy and safety across a wide age range, affirming its potential as a crucial tool in reducing dengue prevalence. Nevertheless, challenges remain, notably the need for comprehensive efficacy and safety data across all serotypes and avoidance of the antibody-dependent enhancement risks seen with previous vaccines. The path forward requires real-world data to confirm the effectiveness and safety of these vaccines, which is a crucial step toward achieving global acceptance and eliminating dengue as a public health concern. However, the vaccine supply and coverage also have critical limitations. Current dengue vaccine manufacturers are unlikely to produce sufficient doses over a subsequent two-year period to achieve satisfactory vaccination rates across the Brazilian population. Consequently, it is imperative to maintain conventional control strategies and amplify preparedness drills to evaluate state and local contingency plans, thereby mitigating the impact of future dengue outbreaks.

Increasing tetravalent vaccination coverage and the implementation of a novel *Ae. aegypti* infection control technologies could reduce the number of dengue cases in Brazil in the coming years. Community engagement through activities such as home cleaning and elimination of potential mosquito breeding sites, facilitated by social media and health education initiatives, must continue to achieve this reduction. Ultimately, a multisectoral approach encompassing sanitary improvements, mosquito control, vaccination, and community mobilization is crucial in the fight against dengue epidemics.
