# Supplementary material for: The greatest Dengue epidemic in Brazil: Surveillance, Prevention, and Control
Source: Rev Soc Bras Med Trop. 2024 Sep 20;57:e00203-2024. doi: 10.1590/0037-8682-0113-2024 (PMC11415067; doi:10.1590/0037-8682-0113-2024)
Supplement: Supplementary file 1 [file 1678-9849-rsbmt-57-e00203-2024-supp1.pdf]

SINAN DENGUE: table of number of cases and deaths in Brazil, from January 2000 to June 15, 2024 by year, month and final classification.

Notification		Probable cases of dengue	Dengue confirmed	Dengue A (Dengue/ Classical)	Dengue B (Warning signs/ complications/ haemorragic fever I and II)	Dengue C (Severe/ Shock syndrome)	Probable deaths of dengue	Confirmed death from dengue	Death under investigation
2000	JAN	14.207	8.806	8.758	48	0	19	19	0
	FEB	24.740	15.196	15.087	109	0	14	14	0
	MAR	32.163	17.580	17.170	410	0	21	21	0
	APR	28.898	16.009	15.979	30	0	16	16	0
	MAY	24.669	15.138	15.113	25	0	8	8	0
	JUN	11.933	6.824	6.815	9	0	9	9	0
	JUL	7.862	4.323	4.314	9	0	5	5	0
	AUG	6.022	3.222	3.217	5	0	4	4	0
	SEP	3.551	1.755	1.753	2	0	4	4	0
	OCT	4.367	2.327	2.320	6	1	2	2	0
	NOV	7.011	3.534	3.512	22	0	4	4	0
	DEC	7.432	4.636	4.608	28	0	5	5	0
2001	JAN	31.479	19.446	19.410	35	1	17	17	0
	FEB	50.508	28.679	28.581	96	2	43	43	0
	MAR	75.367	42.519	42.339	178	2	52	52	0
	APR	93.455	55.137	54.938	198	1	56	56	0
	MAY	97.888	62.372	62.096	276	0	61	61	0
	JUN	45.988	28.074	27.849	224	1	26	26	0
	JUL	24.622	13.780	13.590	190	0	18	18	0
	AUG	16.016	8.600	8.525	74	1	10	10	0
	SEP	10.496	4.962	4.935	27	0	9	9	0
	OCT	12.340	6.138	6.108	28	2	10	10	0
	NOV	13.757	7.543	7.486	56	1	16	16	0
	DEC	16.674	9.373	9.306	67	0	22	22	0
2002	JAN	96.290	57.991	56.973	1.014	4	82	82	0
	FEB	222.672	121.528	119.450	2.074	4	156	156	0
	MAR	276.390	144.118	142.173	1.931	14	172	172	0
	APR	145.382	76.842	75.896	938	8	118	118	0
	MAY	58.981	34.415	33.975	436	4	53	53	0
	JUN	23.028	12.713	12.516	195	2	32	32	0
	JUL	14.663	7.359	7.238	120	1	18	18	0
	AUG	11.976	5.526	5.363	161	2	26	26	0
	SEP	8.796	3.823	3.686	137	0	19	19	0
	OCT	9.245	4.295	4.194	101	0	14	14	0
	NOV	13.887	5.926	5.802	122	2	35	35	0
	DEC	15.783	6.804	6.646	157	1	37	37	0
2003	JAN	35.291	18.554	18.147	402	5	50	50	0
	FEB	71.116	40.842	40.047	783	12	75	75	0
	MAR	89.553	51.626	50.932	675	19	98	98	0
	APR	76.166	42.057	41.501	550	6	83	83	0
	MAY	51.570	28.630	28.150	475	5	45	45	0
	JUN	30.018	15.919	15.727	192	0	34	34	0
	JUL	16.679	8.582	8.471	108	3	34	34	0
	AUG	10.510	5.659	5.599	58	2	21	21	0
	SEP	8.971	4.838	4.792	45	1	18	18	0
	OCT	7.230	2.984	2.933	51	0	21	21	0

SINAN DENGUE: table of number of cases and deaths in Brazil, from January 2000 to June 15, 2024 by year, month and final classification.

Notification		Probable cases of dengue	Dengue confirmed	Dengue A (Dengue/ Classical)	Dengue B (Warning signs/ complications/ haemorragic fever I and II)	Dengue C (Severe/ Shock syndrome)	Probable deaths of dengue	Confirmed death from dengue	Death under investigation
	NOV	8.415	3.150	3.092	58	0	17	17	0
	DEC	11.090	4.694	4.621	71	2	28	28	0
2004	JAN	13.868	6.174	6.084	89	1	22	22	0
	FEB	18.874	8.295	8.192	101	2	26	26	0
	MAR	27.891	13.146	12.987	158	1	48	48	0
	APR	16.568	8.512	8.394	118	0	28	28	0
	MAY	13.126	7.168	7.062	106	0	20	20	0
	JUN	7.720	3.970	3.920	49	1	28	28	0
	JUL	5.527	2.365	2.327	37	1	13	13	0
	AUG	5.042	1.938	1.911	27	0	15	15	0
	SEP	4.414	1.432	1.411	21	0	8	8	0
	OCT	4.689	1.720	1.701	19	0	11	11	0
	NOV	9.003	3.931	3.898	33	0	23	23	0
	DEC	10.145	4.856	4.814	42	0	17	17	0
2005	JAN	15.564	7.987	7.917	70	0	15	15	0
	FEB	22.225	10.927	10.803	122	2	40	40	0
	MAR	37.975	17.663	17.482	179	2	40	40	0
	APR	40.319	19.015	18.773	238	4	48	48	0
	MAY	37.430	19.378	19.049	328	1	37	37	0
	JUN	26.656	14.119	13.875	243	1	34	34	0
	JUL	21.759	11.580	11.314	265	1	41	41	0
	AUG	18.147	9.739	9.561	176	2	27	27	0
	SEP	10.751	5.107	5.029	78	0	22	22	0
	OCT	7.853	3.122	3.071	51	0	17	17	0
	NOV	10.584	4.742	4.693	47	2	17	17	0
	DEC	12.238	5.499	5.419	80	0	36	36	0
2006	JAN	29.811	14.220	13.941	275	4	68	68	0
	FEB	41.464	20.874	20.556	314	4	48	48	0
	MAR	72.342	38.444	38.099	340	5	74	74	0
	APR	85.193	51.253	50.774	476	3	102	102	0
	MAY	68.930	40.852	40.314	531	7	100	100	0
	JUN	32.616	18.334	17.990	340	4	62	62	0
	JUL	19.220	10.727	10.534	191	2	28	28	0
	AUG	14.297	6.995	6.834	159	2	44	44	0
	SEP	8.187	3.769	3.672	96	1	47	47	0
	OCT	8.473	3.566	3.491	75	0	28	28	0
	NOV	13.288	5.955	5.865	89	1	38	38	0
	DEC	17.201	8.360	8.268	88	4	41	41	0
2007	JAN	49.543	21.397	21.156	236	5	15	15	0
	FEB	85.627	41.847	41.452	388	7	22	22	0
	MAR	147.095	77.018	76.326	676	16	42	42	0
	APR	148.785	77.567	76.812	738	17	54	54	0
	MAY	104.646	54.379	53.450	917	12	61	61	0
	JUN	45.514	22.542	21.878	657	7	29	29	0
	JUL	29.876	14.414	13.840	568	6	17	17	0
	AUG	19.514	9.221	8.936	284	1	18	18	0

SINAN DENGUE: table of number of cases and deaths in Brazil, from January 2000 to June 15, 2024 by year, month and final classification.

Notification		Probable cases of dengue	Dengue confirmed	Dengue A (Dengue/ Classical)	Dengue B (Warning signs/ complications/ haemorragic fever I and II)	Dengue C (Severe/ Shock syndrome)	Probable deaths of dengue	Confirmed death from dengue	Death under investigation
	SEP	13.475	5.148	4.927	218	3	18	18	0
	OCT	19.228	5.986	5.738	247	1	7	7	0
	NOV	24.845	7.320	6.919	397	4	22	22	0
	DEC	28.949	9.453	8.842	607	4	20	20	0
2008	JAN	59.784	25.831	23.768	2.042	21	55	55	0
	FEB	74.333	34.712	32.025	2.665	22	65	65	0
	MAR	170.146	75.784	69.365	6.391	28	138	138	0
	APR	331.341	145.789	138.497	7.264	28	154	154	0
	MAY	147.778	73.838	70.212	3.612	14	81	81	0
	JUN	53.312	25.869	24.485	1.372	12	46	46	0
	JUL	19.981	7.762	7.258	503	1	19	19	0
	AUG	11.659	3.727	3.514	211	2	12	12	0
	SEP	8.691	2.629	2.501	126	2	5	5	0
	OCT	10.411	3.851	3.696	155	0	8	8	0
	NOV	13.545	4.738	4.578	159	1	7	7	0
	DEC	18.343	6.277	6.079	196	2	7	7	0
2009	JAN	38.961	17.556	16.933	616	7	19	19	0
	FEB	69.558	35.516	34.456	1.049	11	45	45	0
	MAR	133.604	65.244	63.286	1.943	15	73	73	0
	APR	109.720	58.751	56.826	1.916	9	49	49	0
	MAY	72.798	42.962	41.223	1.732	7	52	52	0
	JUN	33.762	16.872	16.015	854	3	26	26	0
	JUL	19.687	8.476	8.092	378	6	9	9	0
	AUG	13.488	6.173	5.934	235	4	17	17	0
	SEP	10.205	4.600	4.413	182	5	16	16	0
	OCT	12.626	6.659	6.455	202	2	9	9	0
	NOV	30.393	18.866	18.383	475	8	17	17	0
	DEC	55.856	36.978	35.996	968	14	48	48	0
2010	JAN	119.830	81.411	79.684	1.714	13	72	72	0
	FEB	183.451	125.017	122.633	2.366	18	105	100	5
	MAR	290.978	199.741	196.563	3.158	20	134	128	6
	APR	253.099	175.518	172.683	2.809	26	123	112	11
	MAY	195.195	128.184	125.480	2.683	21	103	93	10
	JUN	85.113	49.340	47.626	1.704	10	73	66	7
	JUL	55.250	28.393	27.146	1.236		48	45	3
	AUG	42.301	19.550	18.835	704	11	35	28	7
	SEP	33.499	12.622	12.192	427	3	33	27	6
	OCT	28.362	10.458	10.181	269	8	20	16	4
	NOV	38.259	15.337	15.041	288	8	16	11	5
	DEC	55.917	23.677	23.263	405	9	15	12	3
2011	JAN	94.107	48.937	48.062	866	9	48	43	5
	FEB	153.489	89.326	87.446	1.863	17	86	81	5
	MAR	218.462	130.105	127.856	2.232	17	104	97	7
	APR	235.929	140.876	138.406	2.453	17	115	107	8
	MAY	181.648	113.096	111.036	2.037	23	119	107	12
	JUN	72.397	42.348	41.338	1.002	8	56	45	11

SINAN DENGUE: table of number of cases and deaths in Brazil, from January 2000 to June 15, 2024 by year, month and final classification.

Notification		Probable cases of dengue	Dengue confirmed	Dengue A (Dengue/ Classical)	Dengue B (Warning signs/ complications/ haemorragic fever I and II)	Dengue C (Severe/ Shock syndrome)	Probable deaths of dengue	Confirmed death from dengue	Death under investigation
2011	JUL	34.555	16.839	16.392	444	3	40	32	8
	AUG	27.516	10.707	10.461	245	1	22	18	4
	SEP	23.496	6.954	6.827	125	2	22	18	4
	OCT	28.294	7.305	7.180	123	2	19	15	4
	NOV	34.253	9.549	9.436	113	0	18	16	2
	DEC	45.865	15.533	15.363	167	3	22	14	8
2012	JAN	69.719	25.247	24.995	251	1	24	17	7
	FEB	87.044	33.708	33.306	401	1	27	26	1
	MAR	145.482	57.851	57.175	666	10	63	55	8
	APR	178.729	78.591	77.798	781	12	63	57	6
	MAY	188.719	87.515	86.674	833	8	66	54	12
	JUN	89.656	38.709	38.262	443	4	48	43	5
	JUL	47.587	20.352	20.069	278	5	34	23	11
	AUG	31.833	13.032	12.806	222	4	19	11	8
	SEP	20.770	7.623	7.477	144	2	17	12	5
	OCT	22.135	7.440	7.252	186	2	25	16	9
	NOV	26.423	10.366	10.199	166	1	24	18	6
	DEC	42.083	20.112	19.806	302	4	31	21	10
2013	JAN	181.447	108.442	107.178	1.249	15	74	67	7
	FEB	290.850	173.199	172.002	1.178	19	122	114	8
	MAR	527.741	335.437	333.904	1.507	26	123	116	7
	APR	538.898	327.754	326.355	1.371	28	136	124	12
	MAY	229.953	131.190	130.498	678	14	92	79	13
	JUN	89.823	45.353	44.963	382	8	59	52	7
	JUL	46.927	22.436	22.208	224	4	48	38	10
	AUG	31.160	13.427	13.204	219	4	43	36	7
	SEP	22.373	8.371	8.232	137	2	32	26	6
	OCT	21.531	6.895	6.807	88	0	15	11	4
	NOV	24.849	7.111	7.013	97	1	25	13	12
	DEC	29.567	9.755	9.612	141	2	34	27	7
2014	JAN	56.791	24.567	24.098	425	44	43	39	4
	FEB	80.242	41.723	41.162	507	54	41	38	3
	MAR	116.387	63.786	62.611	1.081	94	62	52	10
	APR	218.551	128.056	124.955	2.938	163	105	93	12
	MAY	186.028	105.162	103.023	2.003	136	86	76	10
	JUN	86.121	44.516	43.664	762	90	64	58	6
	JUL	48.748	21.829	21.326	447	56	40	37	3
	AUG	32.189	12.756	12.508	203	45	33	24	9
	SEP	33.856	15.553	15.339	177	37	33	25	8
	OCT	36.731	16.091	15.920	142	29	36	26	10
	NOV	33.707	14.234	14.070	133	31	19	16	3
	DEC	37.268	16.361	16.186	153	22	23	16	7
	JAN	100.663	62.696	61.803	782	111	61	59	2
	FEB	223.393	148.343	145.581	2.585	177	122	118	4
	MAR	521.693	330.644	324.000	6.282	362	222	209	13
	APR	545.846	340.153	334.057	5.683	413	249	224	25

SINAN DENGUE: table of number of cases and deaths in Brazil, from January 2000 to June 15, 2024 by year, month and final classification.

Notification		Probable cases of dengue	Dengue confirmed	Dengue A (Dengue/ Classical)	Dengue B (Warning signs/ complications/ haemorragic fever I and II)	Dengue C (Severe/ Shock syndrome)	Probable deaths of dengue	Confirmed death from dengue	Death under investigation
2015	MAY	395.894	232.516	229.163	3.039	314	178	162	16
	JUN	169.466	90.659	89.381	1.135	143	82	73	9
	JUL	77.465	35.351	34.711	567	73	50	37	13
	AUG	49.210	20.259	19.936	288	35	23	20	3
	SEP	41.540	17.303	17.019	238	46	38	29	9
	OCT	46.052	18.119	17.807	269	43	28	19	9
	NOV	79.277	31.795	31.320	432	43	37	21	16
	DEC	147.561	62.308	61.292	941	75	61	38	23
2016	JAN	302.334	154.499	152.655	1.712	132	109	77	32
	FEB	537.245	267.748	265.067	2.426	255	255	181	74
	MAR	606.905	289.572	287.013	2.297	262	271	185	86
	APR	422.945	214.413	212.879	1.378	156	229	144	85
	MAY	188.279	94.454	93.665	712	77	104	68	36
	JUN	70.090	30.376	30.019	325	32	47	23	24
	JUL	35.811	13.740	13.571	147	22	34	15	19
	AUG	26.453	8.881	8.746	114	21	30	14	16
	SEP	20.422	5.865	5.730	107	28	26	14	12
	OCT	23.116	5.642	5.505	124	13	19	6	13
	NOV	29.616	7.505	7.306	185	14	24	8	16
	DEC	34.804	8.908	8.669	214	25	26	6	20
2017	JAN	57.270	16.874	16.479	358	37	27	16	11
	FEB	64.307	20.938	20.571	335	32	34	19	15
	MAR	83.252	28.804	28.289	476	39	31	21	10
	APR	73.713	26.319	25.815	458	46	41	30	11
	MAY	66.279	25.386	24.882	457	47	45	39	6
	JUN	41.386	15.265	14.914	306	45	30	19	11
	JUL	21.803	7.605	7.484	103	18	21	10	11
	AUG	19.254	5.818	5.717	80	21	23	18	5
	SEP	20.114	4.500	4.432	57	11	8	7	1
	OCT	23.979	5.137	5.043	80	14	16	10	6
	NOV	22.883	5.224	5.142	67	15	22	10	12
	DEC	24.243	5.734	5.628	87	19	24	9	15
2018	JAN	42.603	13.731	13.432	273	26	12	10	2
	FEB	44.914	16.918	16.389	487	42	26	24	2
	MAR	60.522	25.350	24.632	665	53	38	25	13
	APR	74.419	35.407	34.614	719	74	50	39	11
	MAY	65.479	31.280	30.438	772	70	43	35	8
	JUN	37.920	16.970	16.444	474	52	41	31	10
	JUL	26.181	10.358	10.069	266	23	21	14	7
	AUG	21.401	8.262	8.059	178	25	17	7	10
	SEP	16.406	5.453	5.340	106	7	11	6	5
	OCT	21.654	7.176	7.030	130	16	14	10	4
	NOV	27.515	10.407	10.239	156	12	12	5	7
	DEC	39.866	17.090	16.736	310	44	25	17	8
	JAN	98.582	53.614	52.441	1.086	87	49	43	6
	FEB	180.747	104.681	102.727	1.809	145	94	84	10

SINAN DENGUE: table of number of cases and deaths in Brazil, from January 2000 to June 15, 2024 by year, month and final classification.

Notification		Probable cases of dengue	Dengue confirmed	Dengue A (Dengue/ Classical)	Dengue B (Warning signs/ complications/ haemorragic fever I and II)	Dengue C (Severe/ Shock syndrome)	Probable deaths of dengue	Confirmed death from dengue	Death under investigation
2019	MAR	298.283	177.399	174.844	2.334	221	112	103	9
	APR	503.165	308.707	304.691	3.729	287	230	197	33
	MAY	566.832	356.928	351.942	4.651	335	242	210	32
	JUN	248.034	145.464	142.619	2.654	191	109	80	29
	JUL	112.414	58.151	56.560	1.488	103	65	45	20
	AUG	60.323	27.042	26.132	837	73	37	25	12
	SEP	45.467	17.333	16.817	475	41	33	14	19
	OCT	44.328	15.922	15.535	363	24	32	20	12
	NOV	46.703	19.171	18.664	468	39	33	18	15
	DEC	57.078	27.332	26.795	492	45	41	19	22
2020	JAN	161.760	94.773	93.127	1.497	149	89	79	10
	FEB	289.466	172.974	170.521	2.245	208	136	121	15
	MAR	312.584	188.660	186.059	2.406	195	152	134	18
	APR	229.339	142.609	140.882	1.602	125	113	96	17
	MAY	166.284	85.009	84.032	878	99	77	61	16
	JUN	107.474	42.327	41.929	352	46	41	24	17
	JUL	68.205	22.633	22.422	178	33	27	19	8
	AUG	36.125	11.063	10.930	115	18	20	14	6
	SEP	29.463	7.644	7.556	73	15	15	10	5
	OCT	26.101	6.303	6.235	57	11	14	8	6
	NOV	28.250	6.820	6.761	50	9	8	5	3
	DEC	40.066	12.195	12.044	125	26	20	12	8
2021	JAN	50.282	18.782	18.631	133	18	16	13	3
	FEB	92.430	39.602	39.197	372	33	20	18	2
	MAR	152.846	68.990	68.377	573	40	38	35	3
	APR	178.827	96.710	95.826	802	82	63	60	3
	MAY	151.107	73.056	72.320	676	60	46	40	6
	JUN	98.645	42.440	41.837	550	53	38	32	6
	JUL	61.920	25.007	24.535	436	36	20	15	5
	AUG	44.688	16.017	15.723	276	18	17	13	4
	SEP	35.613	11.061	10.833	204	24	19	15	4
	OCT	29.812	9.061	8.862	180	19	10	5	5
	NOV	38.804	13.775	13.591	171	13	12	8	4
	DEC	75.385	33.383	32.870	469	44	34	23	11
2022	JAN	42.242	37.296	36.590	665	41	26	23	3
	FEB	73.130	65.073	63.524	1.440	109	55	54	1
	MAR	229.741	211.047	207.662	3.115	270	207	202	5
	APR	377.629	348.725	343.608	4.751	366	305	296	9
	MAY	338.314	304.685	299.417	4.871	397	278	267	11
	JUN	129.621	109.819	108.172	1.511	136	95	85	10
	JUL	63.128	52.362	51.623	683	56	34	29	5
	AUG	40.177	32.611	32.125	441	45	23	21	2
	SEP	24.723	20.400	20.044	324	32	24	22	2
	OCT	20.648	16.950	16.622	299	29	22	19	3
	NOV	23.940	19.359	19.102	231	26	19	15	4
	DEC	30.584	25.023	24.667	323	33	27	18	9

SINAN DENGUE: table of number of cases and deaths in Brazil, from January 2000 to June 15, 2024 by year, month and final classification.

Notification		Probable cases of dengue	Dengue confirmed	Dengue A (Dengue/ Classical)	Dengue B (Warning signs/ complications/ haemorragic fever I and II)	Dengue C (Severe/ Shock syndrome)	Probable deaths of dengue	Confirmed death from dengue	Death under investigation
2023	JAN	56.548	50.058	49.397	606	55	37	35	2
	FEB	123.028	108.298	106.874	1.279	145	85	81	4
	MAR	295.858	261.455	257.953	3.252	250	226	209	17
	APR	389.776	332.917	327.566	4.940	411	298	286	12
	MAY	302.129	267.455	263.153	4.031	271	217	200	17
	JUN	103.992	90.009	88.038	1.849	122	93	88	5
	JUL	41.713	35.264	34.175	1.023	66	61	52	9
	AUG	27.955	22.503	21.891	567	45	27	25	2
	SEP	21.152	16.348	15.974	345	29	20	20	0
	OCT	24.567	18.885	18.520	337	28	17	16	1
	NOV	38.443	29.448	28.868	539	41	39	34	5
	DEC	83.781	65.147	63.711	1.352	84	71	50	21
2024	JAN	345.866	270.708	264.983	5.376	349	294	252	42
	FEB	1.018.600	748.032	731.859	15.232	941	835	682	153
	MAR	1.620.683	1.185.272	1.161.958	21.394	1.920	1.832	1.359	473
	APR	1.643.416	1.214.937	1.192.377	20.475	2.085	1.990	1.114	876
	MAY	1.047.704	728.745	712.043	15.391	1.311	1.563	480	1.083
	JUN	151.862	79.178	76.843	2.150	185	255	19	236
